# Sputtered Piezoelectric AlN Thin Films: Parameter Optimisation, Deposition Challenges, and Emerging Perspectives—A Review

**DOI:** 10.3390/mi17080919

**Published:** 2026-07-30

**Authors:** Rangaraajan Muralidaran, Paritosh Dubey, Kuldeep Singh Gour, Shuvam Pawar, Vinod Belwanshi, Jacopo Iannacci

**Affiliations:** 1 Department of Electronics and Communication Engineering, National Institute of Technology Warangal, Hanamkonda 506004, India; rangan2004@gmail.com (R.M.); pawarshuvam@gmail.com (S.P.); 2 Advanced Materials and Corrosion Division, CSIR-National Metallurgical Laboratory, Jamshedpur 831007, India; paritosh.nml@csir.res.in (P.D.); kuldeep.nml@csir.res.in (K.S.G.); 3 Engineering Sciences, Academy of Scientific and Innovative Research, Ghaziabad 201002, India; 4 Materials Engineering Division, CSIR-National Metallurgical Laboratory, Jamshedpur 831007, India; 5 School of Physics and Astronomy, University of Glasgow, Glasgow G12 8QQ, UK; 6 Center for Sensors and Devices (SD), Fondazione Bruno Kessler (FBK), 38123 Trento, Italy

**Keywords:** reactive magnetron sputter deposition, AlN thin films, piezoelectric materials, RF-MEMS, 6G, Silicon Carbide

## Abstract

This article reviews the reactive magnetron sputtering of piezoelectric Aluminium Nitride (AlN) thin films, with a focus on process parameter optimisation and system-level deposition challenges. AlN is a leading material for MEMS and RF applications owing to its c-axis (002) orientation, high acoustic velocity, wide bandgap (∼6.2 eV), and CMOS compatibility. We review the influence of sputtering power, nitrogen flow ratio, substrate temperature, and target-to-substrate distance on crystallographic quality and document practical hardware challenges, including vacuum leakage, grounding faults, target erosion, and mass flow controller drift, that critically affect reproducibility but are systematically underreported in the literature. A perspective is provided on emerging application domains where optimised AlN films address current performance gaps, including next-generation RF/telecom systems towards 6G and Future Networks, harsh environment sensing and actuation, biomedical ultrasound, and IoT energy harvesting. The complementarity between AlN and Silicon Carbide (SiC) is discussed for high-temperature, high-power, and radiation-hard MEMS, where AlN/SiC heterostructures combine the piezoelectric activity of AlN with the mechanical and chemical robustness of SiC. It also incorporates a discussion of dopant- and heteroepitaxy-based AlN engineering, AlN deposition on a wider range of substrates, the role of seed and electrode underlayers, and pulsed-DC sputtering as a third power supply mode alongside RF and conventional DC.

## 1. Introduction

Piezoelectric materials [1] are types of materials that can generate an electrical charge when subjected to mechanical stress, i.e., they can interconvert between electrical and mechanical energy. This phenomenon is known as the piezoelectric effect, which is essential for various applications like sensors, actuators, and energy harvesting devices.

Recent advancements in microfabrication have led to the increased use of piezoelectric thin films in miniaturised systems, particularly in MEMS and RF communication platforms [2]. Thin-film piezoelectrics allow for greater design flexibility, lower power consumption, and integration into semiconductor-based devices. The creation of lead-free, CMOS-compatible piezoelectric materials that preserve electromechanical coupling without posing environmental risks is a key area of current research.

The role of piezoelectric MEMS extends well beyond consumer electronics. First, the transition from 5G to Beyond-5G (B5G), 6G, and Future Networks (FN) is expected to amplify performance indicators such as data rate, latency, and reliability by two to three orders of magnitude relative to 5G while extending operation into the (sub-)THz domain [3]. This demands radio-frequency (RF) passive component filters, switches, attenuators, resonators, and reconfigurable matching networks with quality factors, linearity, and bandwidth that are difficult to attain with purely electronic technologies. RF-MEMS based on piezoelectric AlN are widely regarded as key enablers in this context [3,4]. Second, the deployment of sensing and actuation in aerospace, automotive, energy, and industrial environments calls for transducers capable of operating reliably under high temperatures, high radiation doses, corrosive media, and intense vibration conditions where conventional silicon and lead zirconate titanate (PZT) MEMS fail [5,6].

In this respect, AlN exhibits a combination of properties that make it suitable for the absence of a phase transition below its melting point, a wide direct bandgap of ∼6.2 eV, high acoustic velocity, low dielectric losses, and intrinsic CMOS compatibility. These properties also place AlN in a natural complementarity with Silicon Carbide (SiC), which has emerged as the leading wide-bandgap semiconductor for harsh environment electronics. SiC offers exceptional thermal conductivity, mechanical robustness, oxidation resistance, and radiation hardness, with reported MEMS operation up to 600 °C [5,6]. However, SiC is not piezoelectric; while it can serve as a structural and substrate material, it cannot directly perform electromechanical transduction. AlN, deposited as a thin film on SiC, bridges this gap. AlN/SiC heterostructures combine the piezoelectric activity of AlN with the chemical and mechanical resilience of SiC and benefit from a low lattice mismatch (∼1%) and closely matched thermal expansion coefficients [7,8]. AlN/4H-SiC and AlN/6H-SiC SAW resonators have been demonstrated as high-temperature wireless sensors operating well above the limits of AlN/Si devices [9,10,11,12].

This review focuses on pure AlN, which remains the preferred material for low-loss, high-Q RF filtering and CMOS-compatible MEMS fabrication.

Among different materials, Aluminium Nitride (AlN) has gained attention due to its superior thermal and chemical stability, non-toxicity, and high acoustic velocity [13]. In thin-film form, AlN enables the fabrication of high-frequency devices such as FBARs and SAW filters. The ability to deposit AlN at moderate temperatures using reactive magnetron sputtering makes it highly attractive for integration into advanced electronic systems. While the material properties of AlN are favourable, achieving the desired film performance requires the tuning of deposition conditions and system parameters [14,15,16].

While reviews compiling general reactive sputtering and parameter trends have been previously documented (e.g., by Iqbal and Mohd-Yasin [17]), this article provides a distinct focus on the practical, system-level hardware challenges that critically affect reproducibility but are systematically underreported in the academic literature and on the placement of AlN within the broader landscape of harsh environment, high-frequency, and AlN/SiC heterostructure-enabled MEMS.

Specifically, this article provides a review of reactive magnetron sputtering parameters for AlN thin films and a perspective on emerging applications in next-generation RF/telecom systems (5G, B5G, and 6G), harsh environment sensing and actuation (including AlN/SiC integration), biomedical ultrasound, and IoT energy harvesting. Section 2 covers deposition fundamentals, Section 3 reviews critical process parameters, Section 4 documents practical system-level challenges, and Section 5 presents a forward-looking perspective on emerging applications.

**Figure 1:**
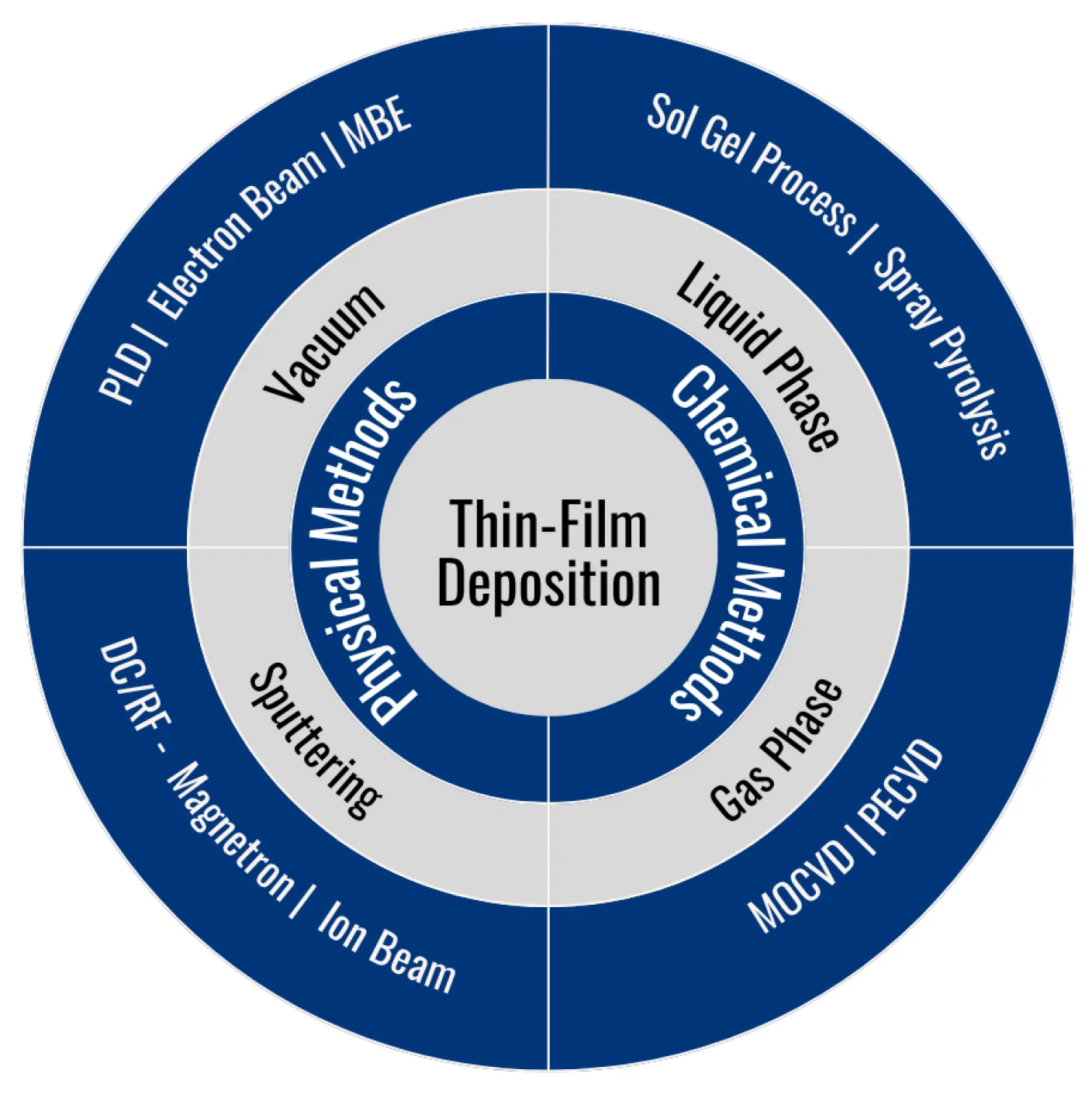
An overview of thin-film deposition methods, categorised into physical and chemical approaches, with representative sub-techniques indicated in the outer ring.

For flexible electronics, AlN films combine high thermal conductivity, high optical transmittance, a low expansion coefficient, and low-temperature preparation. AlN films can be prepared by various techniques such as CVD, reactive magnetron sputtering, reactive evaporation, MBE, ion beam-assisted deposition, laser and plasma-assisted CVD, and MOCVD (Figure 1). Magnetron sputtering [18,19] has been widely used in the industrial process of thin-film deposition over the last few decades due to its high rate, low cost, low temperature, and ease of scaling.

## 2. Deposition Techniques and Sputtering Principles

**Figure 2:**
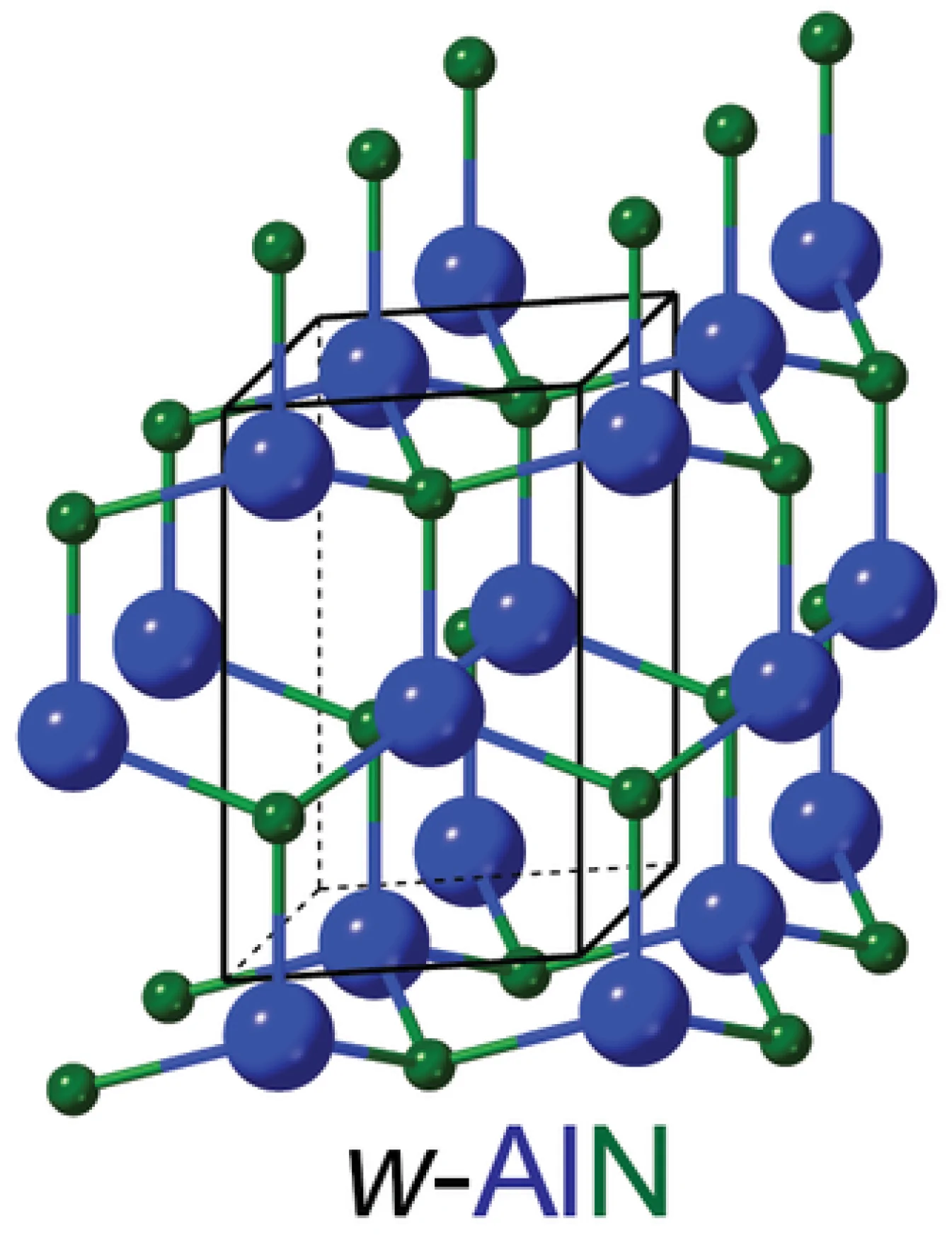
Wurtzite crystal structure of AlN. Adapted from [23].

Aluminium Nitride (AlN) is a III-V compound semiconductor that crystallises in a wurtzite hexagonal structure (Figure 2), characterised by a lack of inversion symmetry, an essential trait for exhibiting piezoelectricity. AlN has a wide bandgap of ∼6.2 eV, making it an excellent insulator with high thermal stability and low electrical losses. It also exhibits high thermal conductivity (∼285 W/mK) and is chemically inert. These attributes make AlN particularly suited for thin-film applications where crystallographic orientation, especially c-axis (002) [20,21] alignment, is crucial for maximising piezoelectric performance [22].

Due to these properties, AlN is widely used in MEMS devices, SAW filters, FBARs, temperature and pressure sensors, and high-frequency RF components. Its CMOS compatibility allows for its integration into silicon-based electronic circuits. AlN is also utilised as a substrate or buffer layer for growing other wide-bandgap semiconductors like GaN. Beyond RF and MEMS, AlN is used in integrated photonics [24]. Its ultra-wide direct bandgap (∼6.2 eV) and transparency across UV to IR make it an excellent candidate for optical waveguides and UV photonics [24].

### 2.1. Sputtering Fundamentals

Sputtering [17,19] and reactive sputter deposition are techniques used in the fabrication of thin films for microelectronics, optoelectronics, and surface engineering. These rely on the interaction of energetic ions with a solid target to excite atoms, which are then deposited onto a substrate to form thin films. Sputtering has the ability to deposit a wide range of materials with controlled thickness, composition, and microstructure. Using reactive gases enables the formation of compound films such as nitrides, oxides, and carbides, expanding the range of materials achievable through PVD [25].

**Figure 3:**
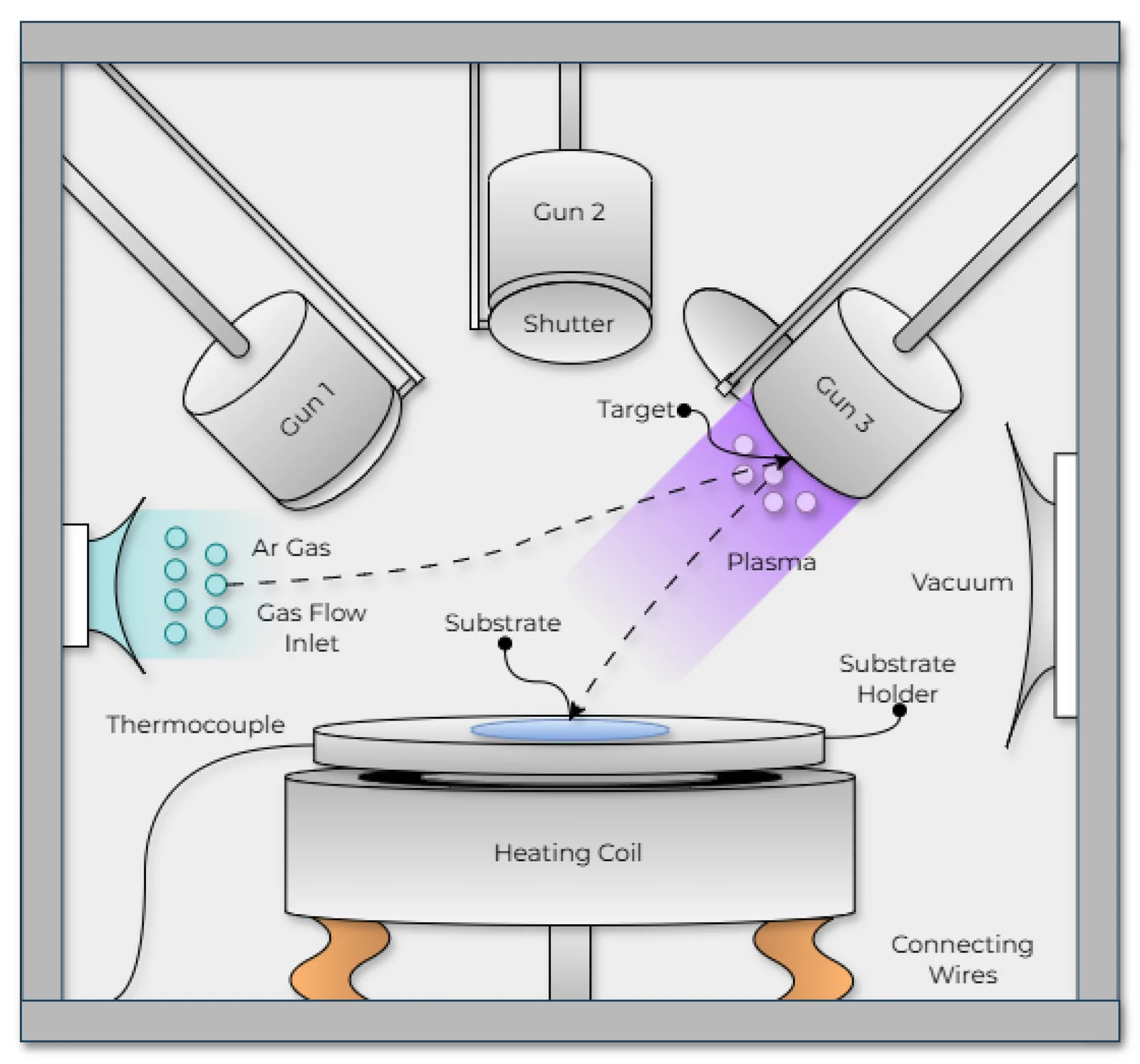
Multi-gun sputter deposition system schematic.

Figure 3 illustrates the key components of a sputter deposition system. The process utilises plasma, which is a partially ionised gas. Within this plasma, free electrons collide with and ionise neutral Ar atoms. The resulting Ar^+^ ions are then accelerated toward the negatively biased target (cathode), initiating the sputtering process.

**Figure 4:**
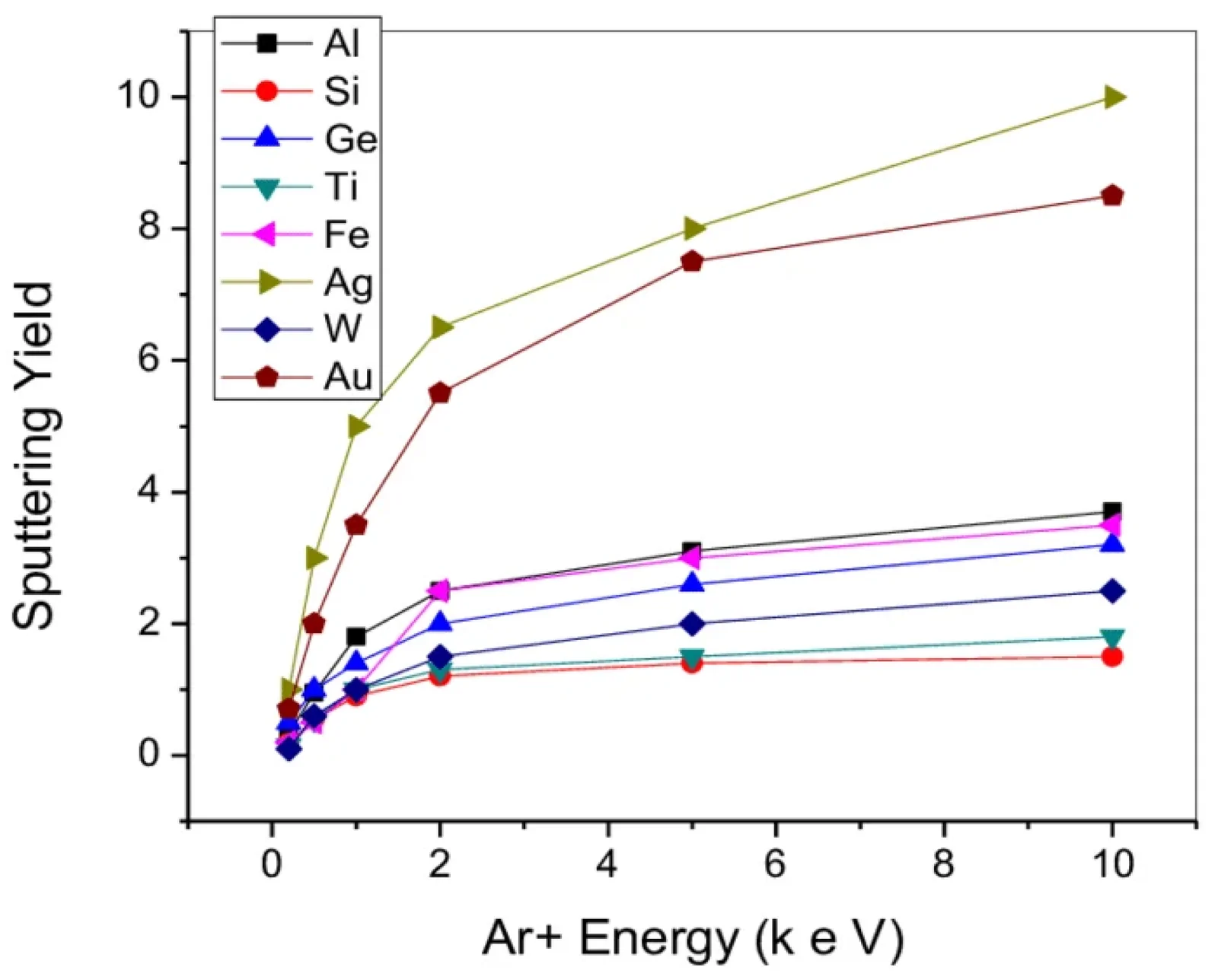
Ar^+^ ion energy vs. sputtering yield. Adapted from [18].

In reactive magnetron sputtering for AlN, the choice of power supply is set by the conduction state of the target rather than simply by the deposited film being insulating. With a metallic Al target in a N_2_/Ar atmosphere, the target surface forms an insulating layer on the cathode (target poisoning). Under conventional DC bias, this charges up, causing arcing and unstable operation. RF power (13.56 MHz) avoids this by reversing the target bias each half-cycle, which is why it is preferred for ceramic AlN targets or heavily nitrided regimes. A third option, pulsed-DC, is compared with RF and DC in Section 2.3. The angular dependence of sputter yield is also notable; increasing the ion angle of incidence up to ∼50° enhances yield due to increased momentum transfer parallel to the surface [18,19,26] (Figure 4).

### 2.2. Reactive Magnetron Sputtering for AlN

**Figure 5:**
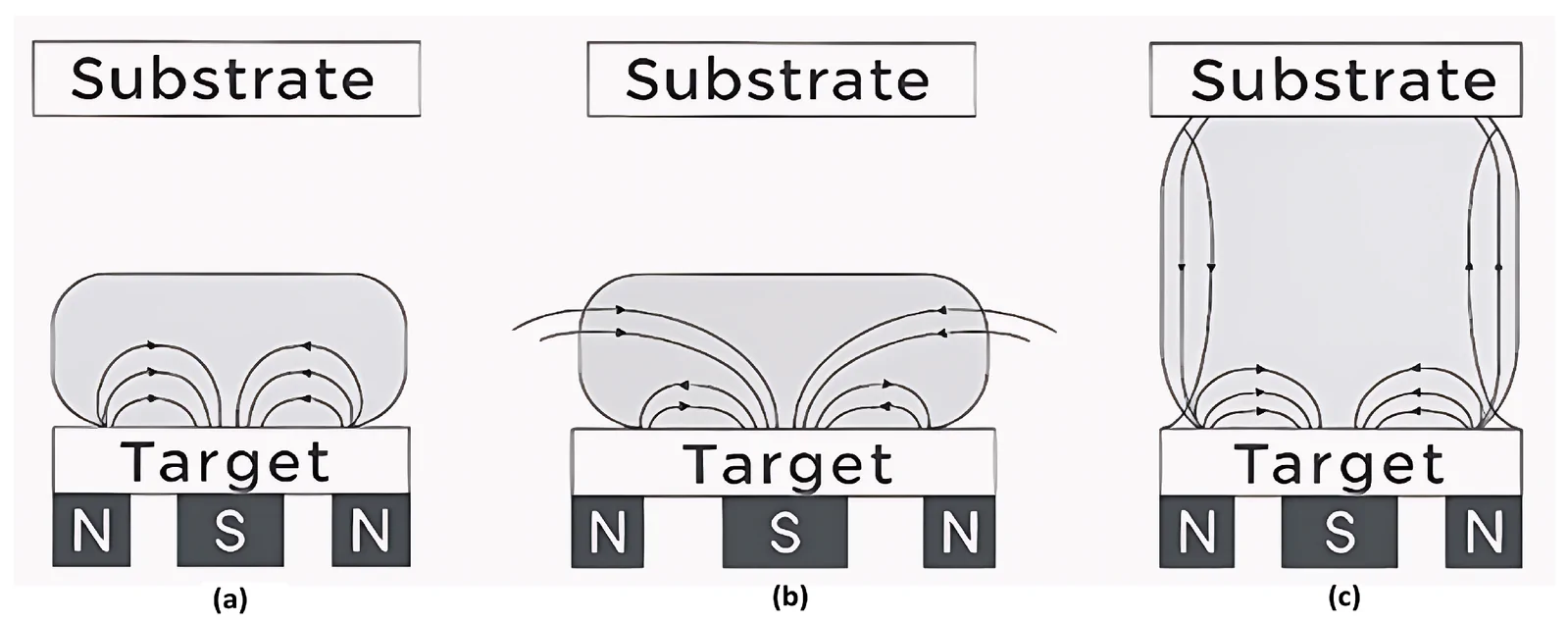
Magnetron sputtering setups: (**a**) balanced; (**b**) Type-1 unbalanced; (**c**) Type-2 unbalanced. Adapted from [27].

Reactive magnetron sputtering involves a static magnetic field parallel to the target surface to trap electrons, increasing ionisation efficiency. Planar magnetrons are used due to their high deposition rates. Figure 5 shows different magnetron configurations. In reactive magnetron sputtering for AlN, nitrogen (N_2_) is introduced alongside argon, and the N_2_/Ar ratio is a critical parameter that governs film stoichiometry and crystallographic orientation, as discussed in Section 3.

### 2.3. Power Supply Comparison: RF, DC, and Pulsed-DC

**Figure 6:**
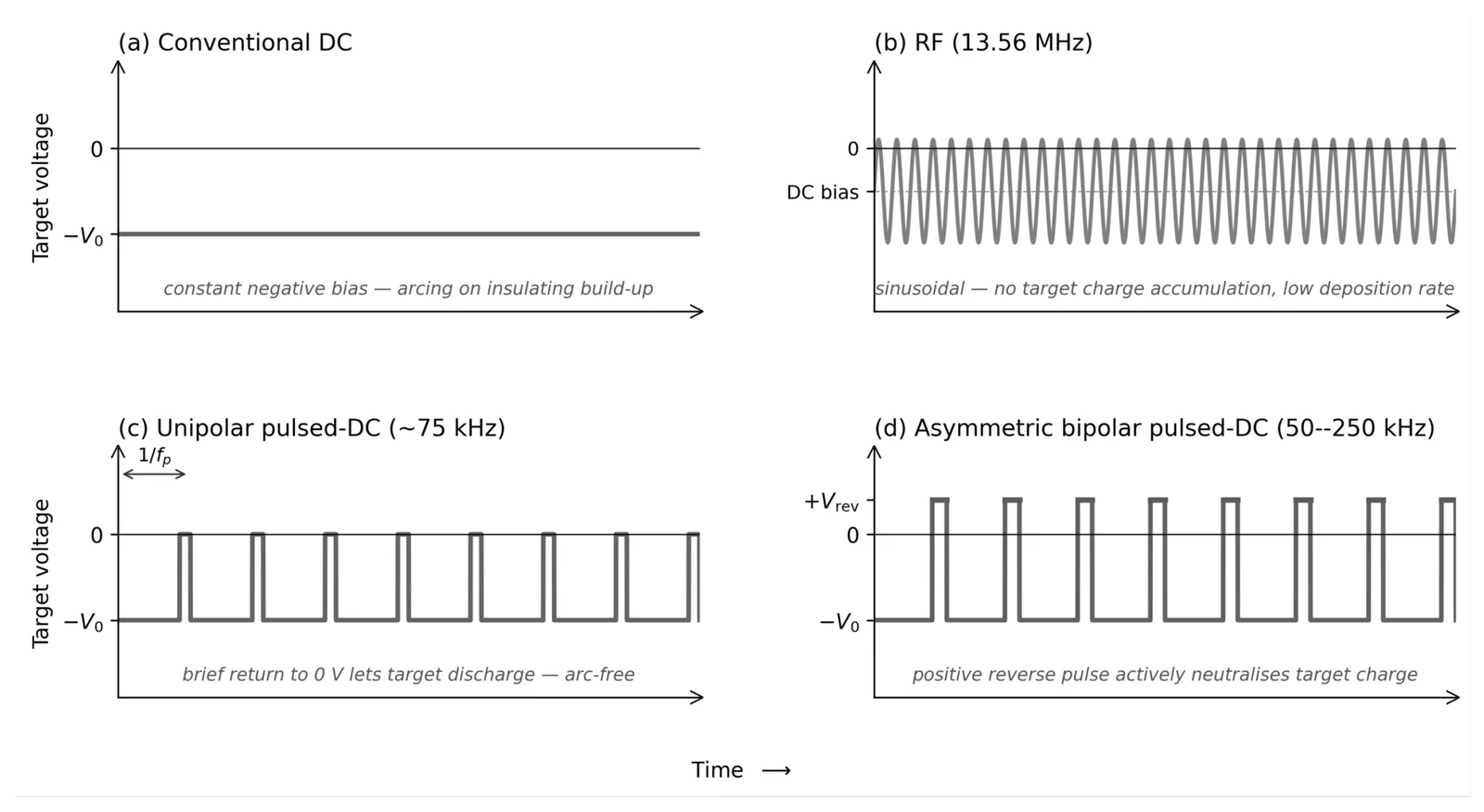
Schematic target voltage waveforms for the three power supply modes discussed in this section: (**a**) conventional DC, where the target sits at a constant negative bias and insulating regions accumulate charge until they arc; (**b**) RF (13.56 MHz), where the fast sinusoidal drive prevents any net target charging but yields the lowest deposition rate; (**c**) unipolar pulsed-DC (∼75 kHz), where a short return to 0 V each cycle allows any accumulated charge to bleed off; and (**d**) asymmetric bipolar pulsed-DC (50–250 kHz), where a brief positive reverse pulse actively drives electrons onto the target to neutralise the built-up positive charge on nitride patches. The vertical axis is target voltage relative to ground; the horizontal axis is time.

RF sputtering (13.56 MHz) sustains stable plasma on fully insulating ceramic AlN targets indefinitely, at the cost of a lower deposition rate and more complex impedance matching hardware. Conventional DC sputtering from a metallic Al target offers the highest deposition rate and simplest hardware but is prone to target poisoning and hysteresis in the metal-to-nitride transition, which degrade reproducibility. Pulsed-DC sputtering, using unipolar pulses (∼75 kHz) or asymmetric bipolar waveforms (50–250 kHz), suppresses arcing by periodically reverse-biasing the target to discharge insulating build-up while retaining DC-like deposition rates. Reported outcomes include a transition from (101) to (002) orientation under applied substrate bias with increasing pulse frequency in the unipolar mode and process windows in which pulsed-DC films match or exceed the crystallinity of RF-sputtered films at substantially lower cost [28] (Figure 6).

## 3. Critical Parameters of AlN Sputtering

### 3.1. Sputtering Power

Across studies, higher sputtering power promotes (002) c-axis orientation, but the power at which this transition occurs varies between systems and is not generally independent from the target material and power supply. Sanjeeva et al. [20] saw a shift from poor, (100)-dominated crystallinity to strong c-axis orientation as DC power rose from only 75 to 175 W on unheated Si(111), using a metallic Al target at a long (130 mm) distance. Sandager et al. [29], on Si(111) at 200 °C with a metallic Al target, instead needed 900–1200 W for high deposition rates, at the cost of higher film stress, with c-axis orientation holding across the 300–1200 W range. A DC study on Mo/Si(111) [30] confirmed a (002) peak from 200 to 500 W, and a wafer-scale study on 8-inch wafers [31] placed the optimum near 8 kW. Liu, H.Y. et al. [32], by contrast, fixed power at a low 145 W and optimised pressure and nitrogen instead, showing that power is not the only route to strong c-axis texture. Liu, W.-S. et al. [33] similarly found that raising power from 300 to 500 W strengthened (002) orientation on their system, which does not define a universal threshold, since the transition depends on target material, throw distance, and the achievable working pressure of the chamber.

### 3.2. Nitrogen-to-Argon Ratio

The N_2_/Ar ratio shows the clearest disagreement across the literature, which we report rather than smoothing it into a single trend. Some studies found that c-axis orientation improves as nitrogen is reduced. Liu, H.Y. et al. [32] saw it increase as nitrogen was reduced from 100% towards 40% (at 0.4 Pa, 145 W), and Sanjeeva et al. [20] found a low N_2_ fraction (25%) sufficient when total flow was also kept low. Others had opposing results: Vashaei et al. [34], with a ceramic AlN target on c-sapphire, saw (002) crystallisation improve as the nitrogen fraction rose across 0–30%, while Khan et al. [35] found that decreasing nitrogen from 60% to 30% degraded c-axis texture and brought in (102)/(103) orientations. Sandager et al. [29] placed the optimum near 50%. We attribute the split mainly to the target poisoning state, as a ceramic AlN target, already fully nitrided, tends to improve with more N_2_, whereas a metallic Al target near the metal-to-poisoned transition can become over-poisoned and destabilised. Liu, W.-S. et al. [33], on their own RF-sputtered system, found that the 100% nitrogen environment gave the highest-intensity (002) orientation across the 50–100% range tested, which is again one system’s behaviour, not a general rule.

### 3.3. Working Pressure

Studies broadly agree that lower working pressure favours c-axis orientation, though the optimal pressure and the residual stress behaviour differ between systems. Lee [36] reported that both sputtering pressure and nitrogen concentration shift the preferred orientation of AlN films. Liu, H.Y. et al. [32] found that c-axis orientation improved as pressure fell (optimum 0.4 Pa). Sandager et al. [29] likewise needed low pressure (0.2 Pa) for high-quality films but reported stress staying tensile across their full 0.1–1.0 Pa range. Panda, Ramaseshan et al. [37], at a larger 140 mm distance and a fixed $5\times 10^{-3}$ mbar, obtained a-axis (100) rather than c-axis (002), indicating that pressure and chamber geometry act together. The base pressure usually quoted in the $10^{-6}$ mbar range is a pre-deposition vacuum figure, distinct from these working pressures.

### 3.4. Substrate Temperature

Substrate temperature shows the most divergence of any parameter here. Liu, W.-S. et al. [33] found a c-axis optimum at 600 °C, with the (002) peak gone by 800 °C through amorphisation. Khan et al. [35], using DC sputtering, reported c-axis quality improving only up to 500 °C, above which orientation shifts to (102)/(103) rather than amorphising; their optimum was 100–200 °C lower. Panda, Ramaseshan et al. [37], by DC sputtering on Si(100) over 35–600 °C at a longer 140 mm distance, found a-axis (100) to be dominant throughout, being the sharpest at 400 °C, so even the orientation family differs from the other two. The reported optima thus span around 300–600 °C and, in one specific case, a different crystal plane entirely (Table 1).

### 3.5. Target-to-Substrate Distance

Iriarte et al. [38] found c-axis orientation to be essentially unaffected by a distance over 30–70 mm, while much larger throw distances (140 mm in Panda, Ramaseshan et al. [37]) gave different results (a-axis rather than c-axis), being more probably from the accompanying pressure, power, and flow differences than from distance itself. We therefore present distance as a system-dependent parameter rather than an independently critical 40–80 mm rule.

### 3.6. Substrate Diversity and Underlying Seed/Electrode Layers

**Figure 7:**
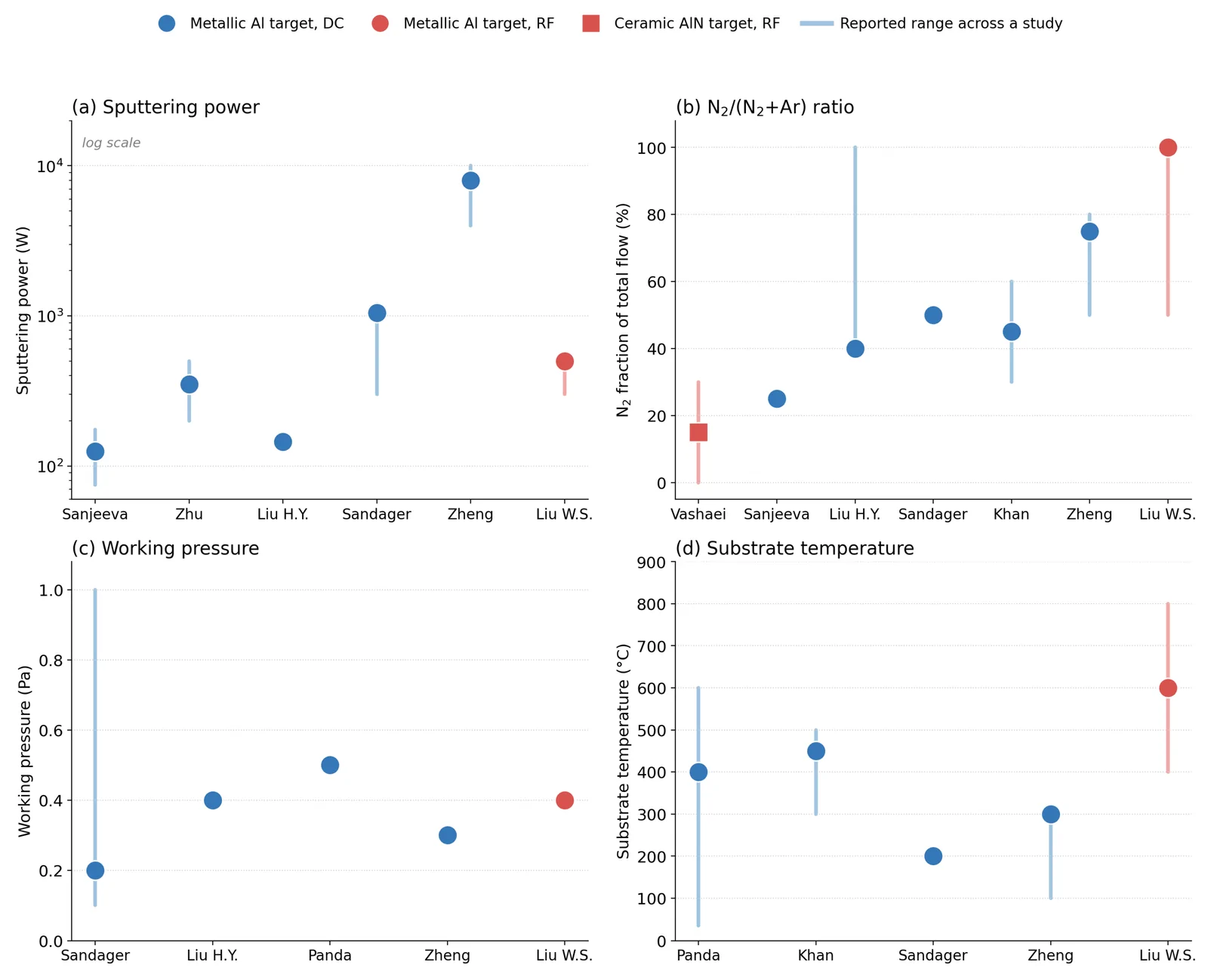
A cross-study comparison of reported optima across independent sputtering studies for the four parameters discussed in Section 3.1, Section 3.2, Section 3.3 and Section 3.4: (**a**) sputtering power on a logarithmic axis; (**b**) N_2_/(N_2_ + Ar) fraction; (**c**) working pressure; (**d**) substrate temperature. Point markers show the reported single value or midpoint of the reported range; vertical bars show the range scanned by each study. Marker shape encodes target material (circle = metallic Al; square = ceramic AlN), and colour encodes power supply (blue = DC; red = RF). The scatter in each panel makes explicit that no single parameter value is universal across systems: apparently similar targets can require substantially different powers, N_2_ fractions, or pressures to reach c-axis (002) orientation, and even the resulting crystal plane is not guaranteed to be (002) in all cases. Studies shown, by panel: (**a**) Sanjeeva [20], Zhu [30], Liu, H.Y. [32], Sandager [29], Zheng [31], Liu, W.-S. [33]; (**b**) Vashaei [34], Sanjeeva [20], Liu, H.Y. [32], Sandager [29], Khan [35], Zheng [31], Liu, W.-S. [33]; (**c**) Sandager [29], Liu, H.Y. [32], Panda [37], Zheng [31], Liu, W.-S. [33]; (**d**) Panda [37], Khan [35], Sandager [29], Zheng [31], Liu, W.-S. [33].

Substrate diversity—Apart from Si and sapphire, AlN has been sputtered directly onto flexible polymer substrates, including polyimide [39] and semicrystalline parylene [40], resulting in flexible piezoelectric cantilevers, pressure sensors, and energy harvesting structures. Reported piezoelectric coefficients on polyimide depend strongly on surface roughening treatment and the presence of a thin Al or Ti buffer layer deposited immediately before AlN. The scatter in reported optima across the power, gas-ratio, pressure, and temperature parameters discussed above is summarised in Figure 7.

Seed and electrode underlayers—AlN is usually grown not on bare substrate but on a metallic bottom electrode (Mo, Pt, Ti, Al, or Ir), and the crystal quality of that electrode propagates into the AlN above. The lattice-matching argument is particularly clean for Mo: the Mo(110) plane has close-packed atomic symmetry with a lattice mismatch of only 1.12% to AlN(002), compared to a 19% mismatch on bare Si(111) [41,42], which is why Zhu et al. [30] were able to obtain a (002) rocking-curve FWHM as low as 0.173° on Mo/Si(111). Akiyama et al. [43] showed that the seed layer under a Mo electrode strongly affects the resulting (002) texture, and a related study on AlN grown directly on Mo confirmed that reactive sputtering can yield strongly c-axis-oriented films on this electrode [44]. Milyutin et al. [45] showed that seed layer choice can set the polarity of sputtered (001) AlN. Pawar et al. [46] compared the three common electrode metals directly, finding that Al, Pt, and Ti each leave a distinct characteristic on the texture, as well as leakage current, with the electrode lattice playing an important role in c-axis growth. Seed and electrode selection should therefore be treated as a process parameter alongside power, pressure, and N_2_/Ar ratio.

## 4. Challenges of AlN Sputtering

**Figure 8:**
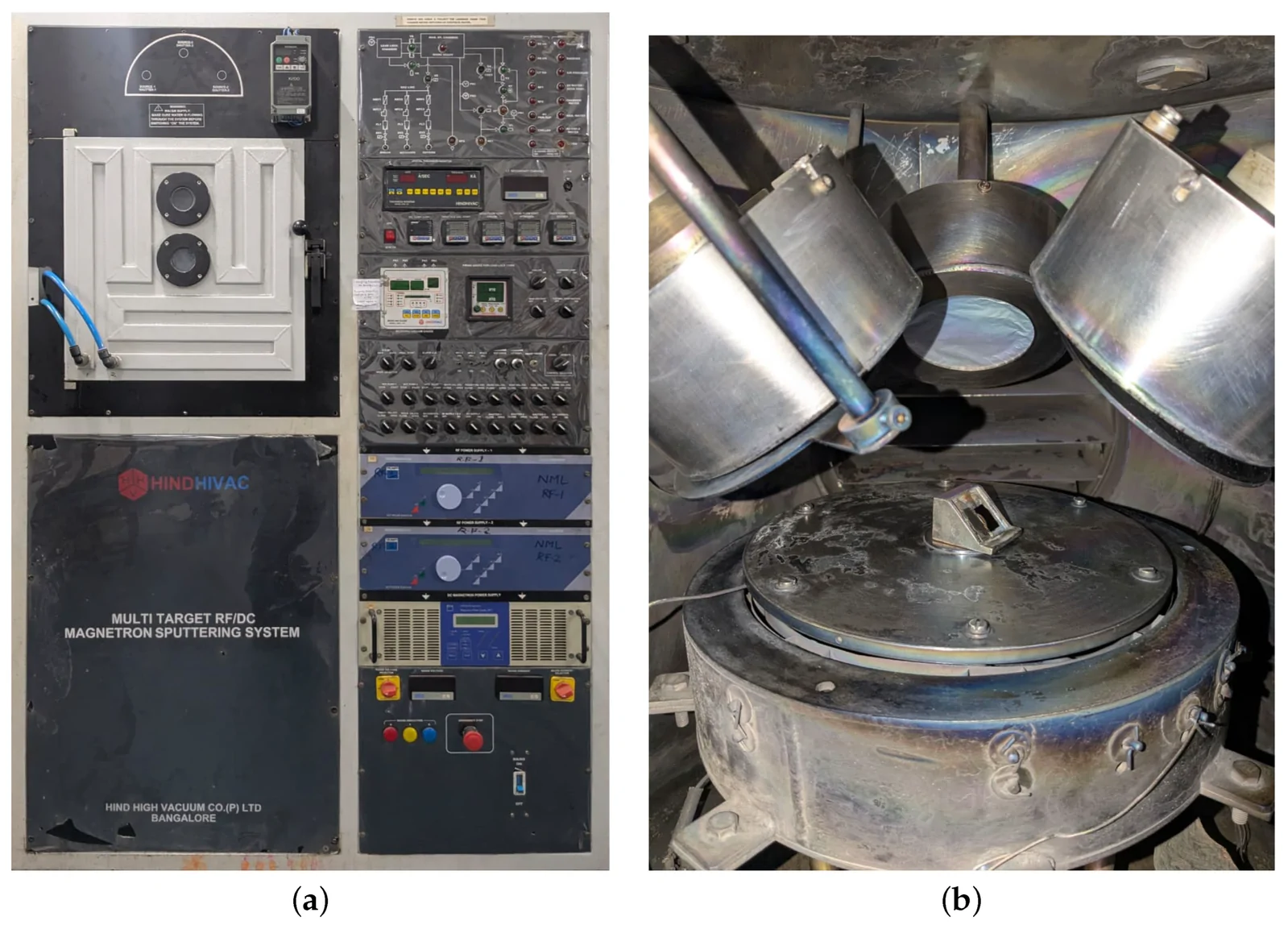
Photographs of authors’ RF magnetron sputtering system at CSIR-National Metallurgical Laboratory, Jamshedpur, India. (**a**) Complete multi-target RF/DC system. (**b**) Close-up of sputter guns and angled substrate holder.

The deposition of c-axis-orientated AlN thin films through reactive magnetron sputtering is governed by process parameters, but real-world implementation frequently encounters system-level limitations that hinder uniform film quality, reproducibility, and electrical performance [17]. Angular deviation between the sputtering target and substrate results in off-normal incidence, leading to non-uniform film growth and shadowing effects. These geometric inconsistencies can disrupt vertical (002) orientation, particularly in co-sputtering systems with fixed-angle guns, such as the authors’ own RF magnetron sputtering system (Figure 8).

A stable high-vacuum environment is essential to suppress moisture contamination. Over time, O-ring degradation, back streaming from oil-based pumps, or undetected microleaks compromise vacuum quality, leading to the unintentional formation of Al–O or Al–O–N phases, degrading the electrical insulation and piezoelectric activity of the film.

Electrical grounding is essential for plasma uniformity and preventing unintentional substrate biasing. Ground faults in substrate heaters or metallic contact points can induce local arcing, unstable sheath formation, or parasitic currents.

Consistent Ar:N_2_ ratios are critical for maintaining stoichiometric AlN deposition. However, mass flow controllers (MFCs) are prone to thermal drift and sensor fouling. These fluctuations can trigger plasma instability or hysteresis in reactive sputtering, leading to undesired transitions between metallic and poisoned modes. Over multiple deposition cycles, metallic residues accumulate on internal chamber walls, shutters, and substrate holders. These deposits can flake off during runs, introducing particulates and surface defects. Preventive maintenance such as shield replacement, physical scraping, or plasma cleaning is required to preserve film purity.

**Figure 9:**
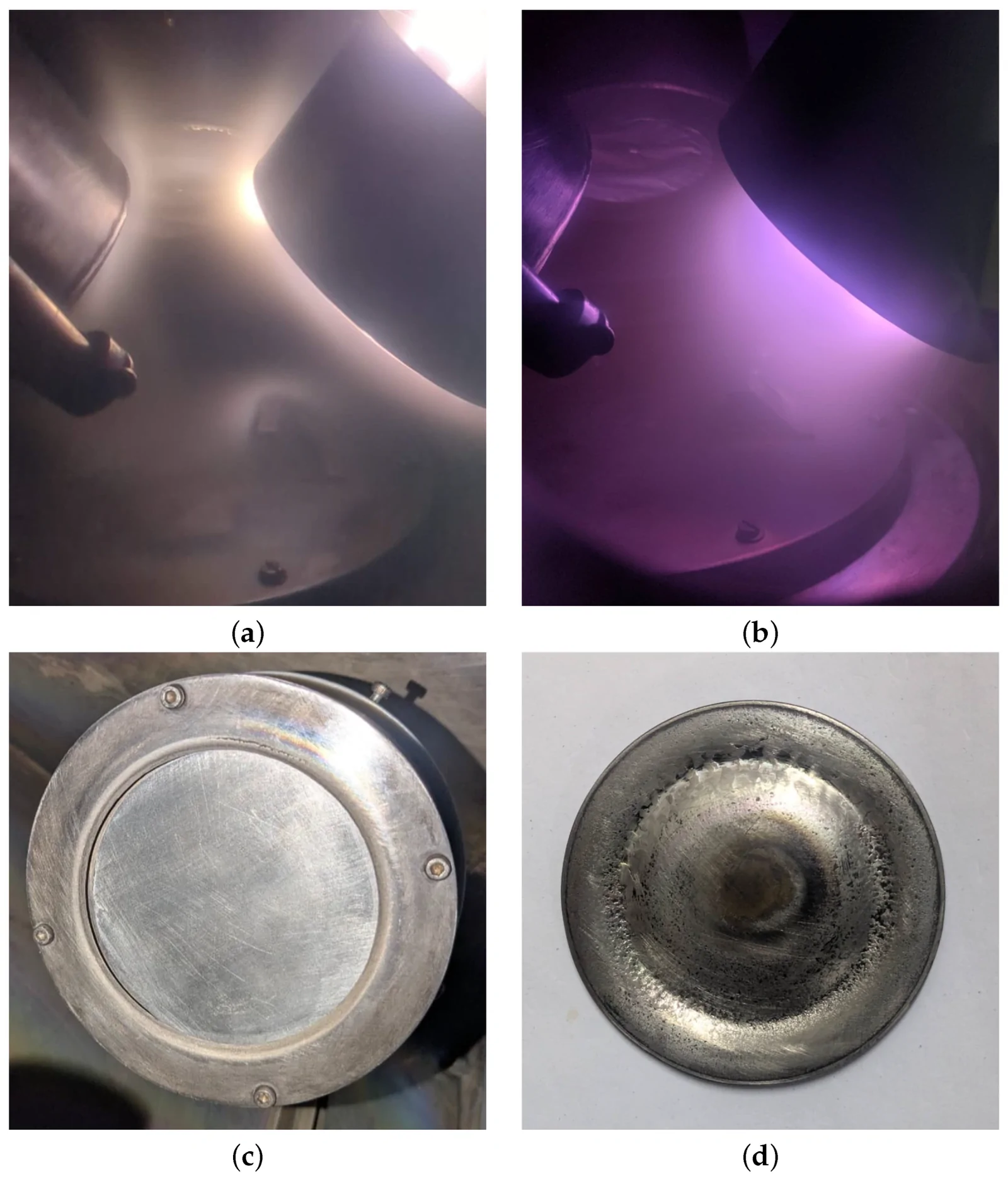
(**a**) Unstable plasma vs. (**b**) stable plasma; (**c**) fresh target vs. (**d**) eroded target showing racetrack erosion profile.

In RF-powered sputtering systems, arcing can occur at electrode interfaces or within improperly matched coaxial lines. This leads to the localised heating of RF cables and electromagnetic interference (EMI). To resolve these issues, using heat-resistant cabling and proper shielding is essential. Unstable plasma conditions and the resulting target erosion are illustrated in Figure 9.

## 5. Perspectives on Emerging Piezo-MEMS Applications

As micro-systems evolve towards higher integration and functionality, the demand for materials that can bridge the gap between electrical and mechanical domains has increased. This is particularly evident in two converging areas: the migration of wireless communications from 5G to Beyond-5G, 6G, and Future Networks (FN) [3,4] and the parallel push for sensors and actuators capable of operating in environments where conventional silicon and PZT-based technologies fail [5,6,11]. In both areas, the limitations of established platforms create distinct opportunities for AlN, either as a stand-alone film or in heterostructures with Silicon Carbide. This section reviews seven application domains where AlN-based devices and, where relevant, AlN/SiC heterostructures address current performance gaps: next-generation RF/telecom filtering and reconfigurable passives towards 6G, biomedical ultrasound, harsh environment sensing and actuation, AlN/SiC heterostructures for extreme environments, IoT energy harvesting, doped/heteroepitaxial AlN, and AlN-based magnetoelectric composites for magnetic field sensing [47].

### 5.1. Next-Generation RF/Telecom: 5G, Beyond-5G and 6G RF Filters

The deployment of 5G New Radio (NR) networks, specifically bands n77, n78, and n79, requires filters with significantly wider bandwidths and higher operating frequencies (>3 GHz). Traditional Surface Acoustic Wave (SAW) devices struggle at these frequencies due to severe acoustic attenuation. AlN-based bulk acoustic wave (BAW) and Lamb wave resonators have emerged as the dominant solution due to AlN’s high phase velocity and CMOS compatibility [48].

However, scaling these devices for 5G and future 6G applications introduces severe deposition challenges. To achieve resonance above 5 GHz, the AlN piezoelectric layer must be scaled down to thicknesses below 400 nm. At this scale, the preferred c-axis orientation constitutes a very small percentage of the total film, severely degrading the electromechanical coupling coefficient ($k_{t}^{2}$).

Looking beyond 5G, the transition towards Beyond-5G (B5G), 6G and Future Networks (FN) is expected to amplify performance indicators by two to three orders of magnitude relative to current 5G targets, with operation extending into the (sub-)THz domain and increased reliance on intelligent, reconfigurable RF front-ends [3,4]. In this scenario, RF-MEMS technologies are widely identified as key enablers, providing high-Q reconfigurable passives such as switches, attenuators, tunable matching networks, and reconfigurable filter banks that cannot be efficiently implemented in pure CMOS at mm-wave and (sub-)THz frequencies [3,4]. Sputtered AlN plays a dual role in this as a piezoelectric layer; it is the imperative material of BAW/FBAR filters and Lamb wave resonators currently dominating 5G front-end modules. Izhar et al. [49] demonstrated a 6G-targeted device, a periodically poled AlScN bulk acoustic wave resonator reaching a $k_{t}^{2}$ of 11.8% and $Q_{p}$ of 236.6 at 17.9 GHz, using a 4-layer piezoelectric film to counteract the coupling and quality factor roll-off that both AlN and AlScN resonators otherwise show above 8 GHz.

### 5.2. Biomedical Ultrasound and Imaging (PMUTs)

Piezoelectric Micromachined Ultrasonic Transducers (PMUTs) based on AlN offer a biocompatible, lead-free alternative to PZT (lead zirconate titanate) that can be monolithically integrated with CMOS circuitry. Because AlN possesses a much lower dielectric constant than PZT, it exhibits a higher piezoelectric voltage constant ($g_{33}$). Large-scale AlN PMUT arrays (e.g., 110 × 56 elements) are capable of imaging epidermal and sub-epidermal layers with resolutions comparable to commercial capacitive sensors [50].

Miniaturised AlN PMUT arrays are being developed for intravascular ultrasound (IVUS) and swallowable diagnostic pills. The primary fabrication challenge for these suspended membrane structures is residual stress management. If the sputtered AlN film exhibits high compressive stress, the PMUT membranes will buckle, altering their resonant frequency and rendering the array non-uniform. Therefore, the precise tuning of the sputtering pressure and RF power, as discussed in Section 3, is mandatory to achieve near-zero or slightly tensile residual stress.

### 5.3. Harsh Environment Sensing and Actuation

Single-crystalline AlN is unique among piezoelectrics for having no phase transition below its melting point and remaining chemically stable up to 1150 °C. This makes it an ideal candidate for aerospace turbine monitoring, deep-well geothermal drilling, and nuclear reactor diagnostics. Conventional PZT sensors fail in these environments due to depoling above their Curie temperature (∼350 °C), and silicon-based MEMS suffer from increased junction leakage and mechanical fatigue at elevated temperatures [5,11].

In this regime, AlN is best understood not as a competitor to wide-bandgap semiconductors such as SiC but as their complement. Pure SiC MEMS, while exceptionally robust mechanically, thermally, and chemically (with operation reported up to 600 °C [5,6]), are not piezoelectric and can suffer from increased leakage currents and a reduced gauge factor at extreme temperatures. AlN, conversely, retains piezoelectric activity well above the Curie limit of PZT but benefits significantly from being deposited on a chemically inert and mechanically stable substrate. This complementarity, rather than competition, is the basis of the AlN/SiC heterostructure platform discussed in Section 5.4.

Recent experiments with flexible AlN thin films on stainless steel diaphragms have demonstrated pressure sensors operating reliably at 900 °C with sensitivities of 0.4–0.5 mV/psi [51]. To realise the full potential of AlN in these environments, the sputtering process must strictly eliminate oxygen contamination in the chamber. Even trace amounts of incorporated oxygen can form aluminium oxynitride phases, which create defect states that exponentially increase leakage currents at high temperatures, effectively shorting the sensor.

### 5.4. AlN/SiC Heterostructures for Extreme Environments

The integration of sputtered AlN thin films on Silicon Carbide (SiC) substrates represents a platform for the next generation of harsh environment MEMS. SiC, as a third-generation wide-bandgap semiconductor, offers high thermal conductivity, a large breakdown field, exceptional radiation hardness, and chemical inertness, making it the material of choice for high-temperature electronics and MEMS structural layers [5,6]. Importantly, SiC is not piezoelectric, so it cannot intrinsically support resonant or acoustic-wave-based transduction. AlN bridges this functional gap.

Several factors make AlN/SiC particularly attractive. The lattice mismatch between AlN and 3C-SiC is small (∼1%), and their thermal expansion coefficients are closely matched, which limits stress-induced cracking during repeated thermal cycling [7,8]. AlN and SiC also enable the design of temperature-compensated electroacoustic devices through appropriate film thickness ratios [8]. Building on this, several groups have demonstrated SAW resonators based on AlN/4H-SiC and AlN/6H-SiC structures with significantly improved high-temperature behaviour compared to AlN/Si. Highly c-axis-oriented sputtered AlN films on 6H-SiC have been reported with FWHM values as low as 0.62°, supporting SAW operation up to 300 °C with stable temperature coefficients of frequency [9], and broader modelling and experimental analyses confirm that wider-bandgap substrates such as 6H-SiC lead to more stable high-temperature behaviour than Si-based equivalents [10]. More recent Pt/AlN/4H-SiC SAW temperature sensors have been investigated for operation in environments where AlN/Si and AlN/sapphire devices are limited by substrate properties [10].

### 5.5. IoT Energy Harvesting

With the increasing use of the Internet of Things (IoT), there is a need for self-sustaining sensor nodes to replace battery-dependent systems, particularly for structural health monitoring and smart agriculture. AlN-based MEMS harvesters have been demonstrated to power leadless pacemakers by harvesting heart vibrations. The challenge with vibration harvesting is that environmental frequencies are typically low (<100 Hz), whereas MEMS devices naturally resonate at high frequencies (kHz to MHz).

The harvested power is not a fixed property of AlN as a material as it depends on the deposition condition of the controlled film quality discussed in Section 3. Residual stress itself is a function of sputtering pressure, and power has been identified as a primary lever. Large residual stress in the AlN active layer degrades the output of cantilever-type vibration harvesters [52,53].

Although pure AlN is standard, Scandium-doped Aluminium Nitride (ScAlN) is becoming popular for high-performance piezo-MEMS to increase the power output at lower frequencies [54]. Doping AlN with scandium (Sc) softens the crystal lattice and increases the piezoelectric charge coefficient ($d_{33}$), thereby increasing the overall output power by up to five times [43,55]. However, the segregation of Sc phases and the necessity for control over doping concentrations pose difficulties for fabrication.

### 5.6. Doped and Heteroepitaxial AlN

The undoped, polycrystalline AlN discussed in Section 2, Section 3 and Section 4 is rarely used in modern devices. Cation doping and heteroepitaxial integration with other III-nitrides are now extending to AlN’s piezoelectric, polarisation, and thermal performance.

Cation doping—Scandium is the most studied dopant, but other cation substitutions have been used both for piezoelectric enhancement and for tuning polarity. Anggraini et al. [56] used Si and MgSi dopants to invert the polarity of sputtered AlN, showing that dopant choice controls polarity, not just piezoelectric magnitude. Doping during reactive sputtering brings its own challenges apart from those in Section 4. Co-sputtering or alloy target erosion has to hold a stable and uniform dopant concentration across the wafer, which sets practical challenges in terms of the achievable enhancement. Gao et al. [57] deposited ScAlN directly onto Si and SiO_2_/AlN/Mo on separate functional layers, reporting a (002) FWHM of 1.7° and a surface roughness of 1.76 nm for the Si-direct film and integrating it into a working film bulk acoustic resonator (FBAR) via wafer-level thin-film transfer.

Heteroepitaxy and polarity control—AlN is also grown heteroepitaxially on GaN and related III-nitride heterostructures [58], where strain-induced polarisation charge and thermal dissipation become of concern. Wolff et al. [59] reported polarisation switching in sputter epitaxial Al_0.92_Sc_0.08_N/GaN heterostructures, which shows that these interfaces are sensitive to growth sequence and chemistry. Maintaining crystallinity, controlling dopant or alloy incorporation, and achieving abrupt, low-defect interfaces are thus the three challenges for doped and heteroepitaxial AlN.

## 6. Conclusions

In this review and perspective article, the deposition of piezoelectric AlN thin films via reactive magnetron sputtering was critically examined with a focus on the impact of process parameters and system-level challenges. AlN has demonstrated significant promise in MEMS and RF applications due to its strong c-axis (002) orientation, thermal stability, and CMOS compatibility.

Across the independent studies reviewed, sputtering power and the nitrogen-to-argon ratio both influence (002) preferential orientation but neither does so via a single universal threshold. The power level and nitrogen fraction that favour c-axis growth depend on target material (metallic Al versus ceramic AlN), target poisoning state, and chamber geometry, and different research groups report power optima spanning two orders of magnitude and nitrogen optima spanning the full 0–100% range. Substrate temperature has been shown to influence atom mobility and surface diffusion, where temperatures around 600 °C were reported to yield the most crystalline films for the specific substrate and system conditions of Liu et al. [33]. The results may vary with substrate type and chamber configuration, and excessive heating generally leads to degradation and amorphisation.

This paper also highlighted several practical equipment-related issues, such as target–substrate misalignment, vacuum leakage, grounding problems, and contamination from metal residues, that can undermine deposition consistency and film performance. Beyond fabrication, the perspective sections placed AlN within the wider technology of next-generation RF/telecom systems (5G, B5G, 6G, and Future Networks) and harsh environment MEMS. In particular, AlN and Silicon Carbide (SiC) were highlighted. SiC provides thermal, mechanical, and radiation robustness but is not piezoelectric, while sputtered AlN supplies the missing electromechanical transduction. AlN/SiC heterostructures, with their low lattice mismatch, matched thermal expansion, and opposite temperature coefficients of delay, are therefore identified as a strategic platform for future high-temperature, high-frequency, and radiation-hard piezo-MEMS [7,8,9,10,11]. In conclusion, the successful realisation of high-quality AlN thin films for piezoelectric applications depends on the understanding of deposition parameters, hardware limitations and specific application constraints, and a system-level view in which AlN is co-designed with substrates and integration tailored to the target application domain.

## Figures and Tables

**Table 1:** Reported substrate temperature optima for c-axis AlN sputtering across independent studies, illustrating system dependence rather than a universal value.

Study	Power Supply/Target	Reported Optimum and Trend	Dominant Orientation
Liu, W.S. et al. [33]	RF, metallic Al target	600 °C optimum; (002) peak vanishes by 800 °C (amorphisation)	(002), c-axis
Khan et al. [35]	DC, metallic Al target	Improves up to 500 °C; above 500 °C shifts to competing planes	(002) below 500 °C; (102)/(103) above
Panda, Ramaseshan et al. [37]	DC, metallic Al target, 140 mm TSD	Sharpest texture at 400 °C; declines above	(100), a-axis (not c-axis)

## Data Availability

Data available on request from the authors.
